# Abnormal intrinsic brain functional network dynamics in patients with cervical spondylotic myelopathy

**DOI:** 10.1007/s11571-022-09807-0

**Published:** 2022-04-29

**Authors:** Guoshu Zhao, Yaru Zhan, Jing Zha, Yuan Cao, Fuqing Zhou, Laichang He

**Affiliations:** 1 Department of Radiology, the First Affiliated Hospital of Nanchang University, No. 17 Yongwaizheng Street, Nanchang, Jiangxi 330006 People’s Republic of China; 2 The 908th Hospital of Chinese People’s Liberation Army Joint Logistic Support Force, Fuzhou, 330006 People’s Republic of China; 3 Department of Nuclear Medicine, West China Hospital of Sichuan University, Chengdu, 610041 People’s Republic of China; 4 Huaxi MR Research Center (HMRRC), Department of Radiology, West China Hospital of Sichuan University, Chengdu, 610041 People’s Republic of China; 5 Neuroimaging Lab, Jiangxi Province Medical Imaging Research Institute, Nanchang, 330006 People’s Republic of China

**Keywords:** Cervical spondylotic myelopathy, Dynamic functional connectivity, Graph theory analysis, Brain functional network

## Abstract

The specific topological changes in dynamic functional networks and their role in cervical spondylotic myelopathy (CSM) brain function reorganization remain unclear. This study aimed to investigate the dynamic functional connection (dFC) of patients with CSM, focusing on the temporal characteristics of the functional connection state patterns and the variability of network topological organization. Eighty-eight patients with CSM and 77 healthy controls (HCs) were recruited for resting-state functional magnetic resonance imaging. We applied the sliding time window analysis method and K-means clustering analysis to capture the dFC variability patterns of the two groups. The graph-theoretical approach was used to investigate the variance in the topological organization of whole-brain functional networks. All participants showed four types of dynamic functional connection states. The mean dwell time in state 2 was significantly different between the two groups. Particularly, the mean dwell time in state 2 was significantly longer in the CSM group than in the healthy control group. Among the four states, switching of relative brain networks mainly included the executive control network (ECN), salience network (SN), default mode network (DMN), language network (LN), visual network (VN), auditory network (AN), precuneus network (PN), and sensorimotor network (SMN). Additionally, the topological properties of the dynamic network were variable in patients with CSM. Dynamic functional connection states may offer new insights into intrinsic functional activities in CSM brain networks. The variance of topological organization may suggest instability of the brain networks in patients with CSM.

## Introduction

Cervical spondylotic myelopathy (CSM) is the most common cause of cervical spinal cord compression, with impaired sensory and motor functions, which seriously affects the quality of life of patients (Yarbrough et al. 2012). Studies have indicated that the spinal cord has several connections with the brain structure and function, and that the high central nervous system can regulate the motor and sensory functions in patients with CSM (Bernabéu-Sanz et al. 2020; Dong et al. 2008; Duggal et al. 2010; Tinnermann et al. 2017). Brain plasticity or reorganization has been reported to affect neurological function recovery by affecting structural and functional changes in the cervical spinal cord (Duggal et al. 2010). Therefore, the underlying mechanism of central nervous system damage in patients with CSM may help explain the differences in surgical outcomes.

Previous neuroimaging studies have clarified impairments in the brain networks of patients with cervical spinal cord injury (Oni-Orisan et al. 2016; Ryan et al. 2018). Zhao et al. (Zhao et al. 2020) found cognitive deficits and their correlation with alterations in graph theory properties and functional connectivity in the sensorimotor network (SMN), default mode network (DMN), and visual network (VN). In addition, our previous study found alterations in the intrinsic functional plasticity within the SMN in patients with CSM (Zhou et al. 2015). Furthermore, we analyzed the disruption of human brain connectivity networks in patients with CSM using graph theory analysis (Cao et al. 2021). However, all these studies treated intrinsic brain activity as stable and invariable in whole-scan time courses, which is called static functional connectivity (sFC). SFC implicitly ignores the functional interaction over time by calculating the statistical correlation between regions during whole-scan time courses. Recently, emerging evidence has shown that functional connectivity between brain regions during scan sessions is highly dynamic (Allen et al. 2014; Damaraju et al. 2014; Di and Biswal 2015; Gonzalez-Castillo et al. 2014; Leonardi et al. 2013).

Many studies have pointed out that dynamic functional connectivity provides new insights for a better understanding of pathophysiological mechanisms in the central nervous system by capturing the transition between different states (Allen et al. 2014), (Li et al. 2020). Liao et al. (Liao et al. 2015) had demonstrated the characteristics of dynamic functional architecture in large-scale human brain networks during resting-state functional magnetic resonance imaging (rs-fMRI) to deepen the understanding of spontaneous human brain network dynamics. In addition, topological characteristics based on graph theory analysis have been regarded as a good way to explore the topological properties of human brain networks (Bullmore and Sporns 2009). Abnormalities of the brain networks were only partial representations of static functional connections, which inadequately represented the working patterns of brain networks in patients with CSM. As mentioned above, we explored sFC networks using the graph theory method; we also discuss the topological properties of dynamic functional connection networks.

Sliding window analysis is a method used for dynamic FC (dFC) analysis. The selection of the window length is a controversial issue (Shakil et al. 2016; Wilson et al. 2015). The most recommended window length is between 30 and 60 s (Li et al. 2018; Shakil et al. 2016). In addition, the K-means clustering method can divide these outcomes within all sliding time windows into several states to better describe the operating mode of the human brain during whole-scan time courses.

Accordingly, to capture the dynamic configurations of brain networks, we hypothesized that patients with CSM would exhibit dynamic interactions between brain networks. Our group examined (i) whether patients with CSM exhibit different patterns of dynamic connectomics compared to healthy controls (HCs); (ii) how the topological organization of brain endogenous functional activity changes on the time scale; and (iii) whether there is a potential relationship between the dFC variability alterations and the clinical characteristics of patients with CSM.

## Materials and Methods

This study was approved by the Institutional Review Board of the First Affiliated Hospital of Nanchang University. All the patients’ consents were also required and obtained.

### Participants

There were 88 patients with CSM (45 males and 43 females; mean age 49.22 ± 7.91 years; range 30–62 years) from the First Affiliated Hospital of Nanchang University and 77 HCs of level-matched age, sex, and education (39 males and 38 females; mean age 45.13 ± 8.76 years; range 25–64 years) were recruited in our study from January 2016 to December 2020. The gold diagnostic standard of CSM (Emery 2001): (1) clinical manifestations of cervical spinal cord injury; (2) radiographically confirmed spinal cord compression; (3) no amyotrophic lateral sclerosis, intramedullary tumors, secondary adhesion arachnoiditis, multiple peripheral neuritis, or spinal cord injury. In addition, patients were required to meet the following inclusion criteria: (1) volunteer to enroll in the study, (2) have clear evidence of cord compression on a cervical spine MRI, and (3) have had no medication therapy or decompression surgery. The exclusion criteria were as follows: (1) having other neurological disorders, (2) a history of psychiatric disorders, and (3) poor cooperation and so much head movement during image scanning. All patients were assessed with the Japanese Orthopaedic Association (JOA) scores (Azimi et al. 2012) and the Neck Disability Index (NDI) (Vernon 2008).

### MRI data acquisition

All participants were scanned with a 3.0 T MRI (Siemens Trio Tim, Erlangen, Germany) scan with an 8-channel head coil. High-resolution anatomic images were acquired using a 3D T1-weighted spoiled gradient recall sequence with the following parameters: TR = 1900 ms, TE = 2.26 ms, flip angle = 9^°^, FOV = 256 × 256 mm, matrix = 256 × 256, slice thickness = 1 mm, number of slices = 176, voxel size = 1.0 × 1.0 × 1.0 mm^3^, and interslice gap = 0.5 mm. The echo-planar imaging (EPI) sequence parameters were as follows: TR/TE = 2000 ms / 30 ms, flip angle = 90^°^, FOV = 200 × 200 mm, matrix = 64 × 64, number of slices = 30, slice thickness = 4 mm, interslice gap = 1.2 mm, voxel size = 3.0 × 3.0 × 4.0 mm^3^, and 240 time points (8 min 6 s).

### Data pre-processing

fMRI preprocessing was performed using the Data Processing Assistant for Resting-State (version 5.0; DPABI, http://www.restfmri.net) (Chao-Gan and Yu-Feng 2010). The procedures were as follows: (1) removal of the first ten time points; (2) slice timing correction; (3) head motion correction; (4) co-registration of functional data to the structural T1-weighted image; (5) normalization into the Montreal Neurological Institute (MNI) space with a resampling voxel size of 3 × 3 × 3 mm^3^; (6) removal of linear trends; (7) band-pass filtering (0.01–0.08 Hz); (8) nuisance covariate regression; (9) smoothing (6-mm full-width half-maximum Gaussian kernel).

### Construction of dynamic functional networks

For each participant, we employed a commonly used sliding time window approach to estimate dFC based on the dynamic brain connectome (DynamicBC) analysis toolbox (Liao et al. 2014). An automated anatomical labeling 90 (AAL) atlas was used to divide the brain into 90 anatomical regions, with a sliding time window of 30 volumes (60 s). This window was shifted in time with a step size of 3 TR (6 s) (Li et al. 2018, 2019; Liao et al. 2018). The choice of window length was based on earlier studies that recommended a range of 30 to 60 s to capture spontaneous fluctuations (Luppi et al. 2021; Preti et al. 2017; Shakil et al. 2016). Pearson’s correlation coefficients were calculated between the time series from all brain regions, resulting in a 90 × 90 matrix per window per subject, with a total of 67 sliding time windows for each subject. Finally, we calculated the standard deviation of the Z value to represent the change in the time series of the correlation coefficient maps and characterize the variability of the dFC.

### Characteristics of dynamic functional networks

K-means clustering analysis was used to conduct normalized graph clustering on the functional connection matrix (Shine et al. 2016). We then extracted the FC time-series signals to calculate the centroid, number of transitions, mean dwell time (MDT), and fractional windows. We then extracted the brain networks of all subjects in each state to further compare differences between groups within the same state using a two-sample t-test (with age, gender, and education as covariates; network-based statistics correction with edge *p* < 0.001, component *p* < 0.05, literation = 1000).

### Graph theory analysis of dynamic functional networks

We extracted the dFC networks under all sliding time windows to further calculate their topological properties using graph-theory-based methods. Ultimately, 11,055 brain networks were identified. All network analyses were performed using the graph theoretical network analysis (GRETNA) toolbox 2.0.0 (http://www.nitrc.org/projects/gretna) (Wang et al. 2015). To generate an undirected and unweighted graph, the threshold range of sparsity was identified as 0.1–0.4, with an interval of 0.01 based on a previous study (Achard and Bullmore 2007). For the dFC network within each sliding time window, we calculated the variance of both the global and nodal network metrics (He et al. 2009; Koshimori et al. 2016; Yu et al. 2015). We then calculated the time-varying variance of the network metrics using a two-sample t-test to explore the topological properties of dynamic brain networks.

### Statistical analysis

Statistical analyses were conducted using GraphPad software. Differences in categorical variables between groups were tested and compared using a chi-squared test, whereas those between continuous variables were evaluated using a two-sample t-test. We then assessed the relationships between the dynamic functional network metrics, JOA scores, and NDI scores in the CSM group.

## Results

### Demographics and clinical characteristics

The demographics and clinical characteristics of the subjects are shown in Table 1. Patients with CSM had a mean symptom duration of 10.24 ± 6.10 months, mean JOA score of 11.10 ± 1.78, and mean NDI score of 0.32 ± 0.09.

**Table 1:** Demographic and clinical characteristics of the subjects

Clinical variables	CSM (n = 88)	HC (n = 77)	*p* value
Age	49.22 ± 7.91	45.13 ± 8.76	0.355
Gender			0.798
Male	45	39	
Female	43	38	
Education (years)	12.91 ± 1.21	12.64 ± 1.20	0.149
Duration of symptoms (month)	10.24 ± 6.10	NA	–
NDI scores	0.32 ± 0.09	NA	–
JOA scores	11.10 ± 1.78	NA	–
Motor upper	2.09 ± 0.47	NA	–
Motor lower	2.23 ± 0.84	NA	–
Sensory deficit	3.24 ± 0.88	NA	–
Bladder dysfunction	2.98 ± 0.15	NA	–

*CSM* cervical spondylotic myelopathy; *HC* healthy control; *NDI* neck disability index; *JOA* Japanese Orthopedic Association

### Temporal characteristics of dynamic functional networks

**Fig. 1:**
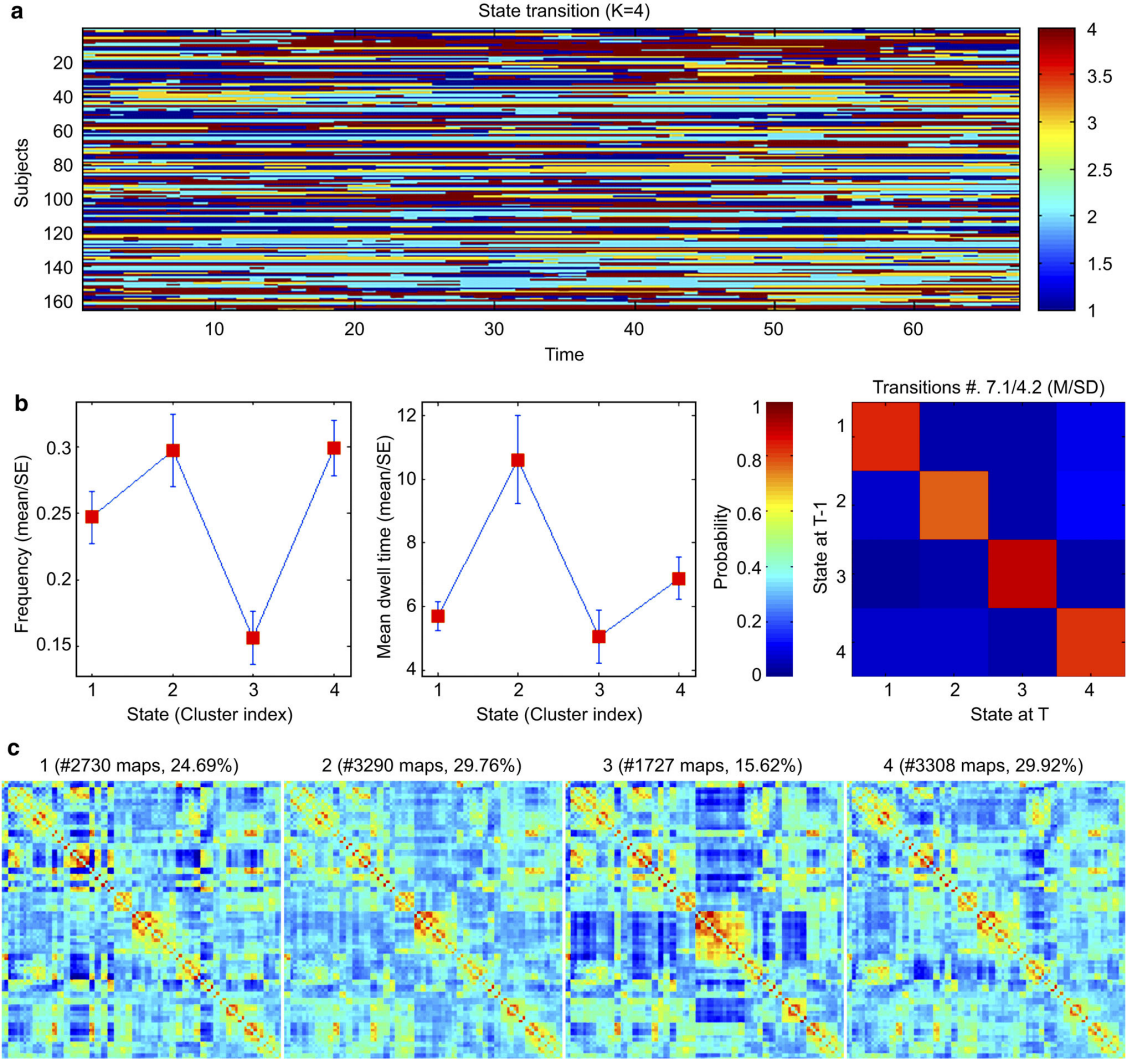
**a** State switching mode of all subjects on all sliding windows; **b** The mean dwell time and the probability of transition between state 1, state 2, state 3, and state 4; c. The frequency of occurrence of state 1, state 2, state 3, and state 4

**Fig. 2:**
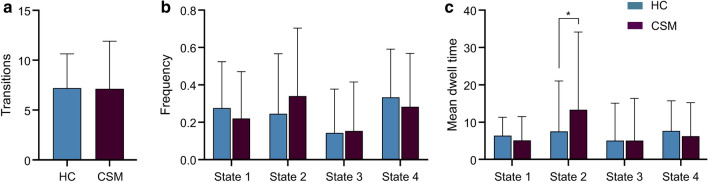
Differences of temporal properties between the two groups. No group differences were found in the number of transitions and state frequency between these four states. The mean dwell time of state 2 was significantly different (mean ± SD for healthy controls: 7.5 ± 13.6; for CSM patients: 13.4 ± 20.8, *p* < 0.05). Purple and blue represent the CSM and HC groups, respectively

According to the above clustering criteria, we obtained the optimal number of clusters K = 4. We determined four types of states during resting-state MRI scans among subjects: state 1 (24.69%), state 2 (29.76%), state 3 (15.62%), and state 4 (29.92%) (Fig. 1). In particular, the MDT in state 2 was significantly longer in the CSM group than in the HC group (mean ± SD for healthy controls: 7.5 ± 13.6; for CSM patients: 13.4 ± 20.8, *p* < 0.05*,* Fig. 2). No group differences were observed in the number of transitions between the four states. We also performed a further analysis of correlations in the CSM group. However, there was no significant correlation between the dFC properties and clinical characteristics.

### Differences between CSM and HC groups within the same state

**Fig. 3:**
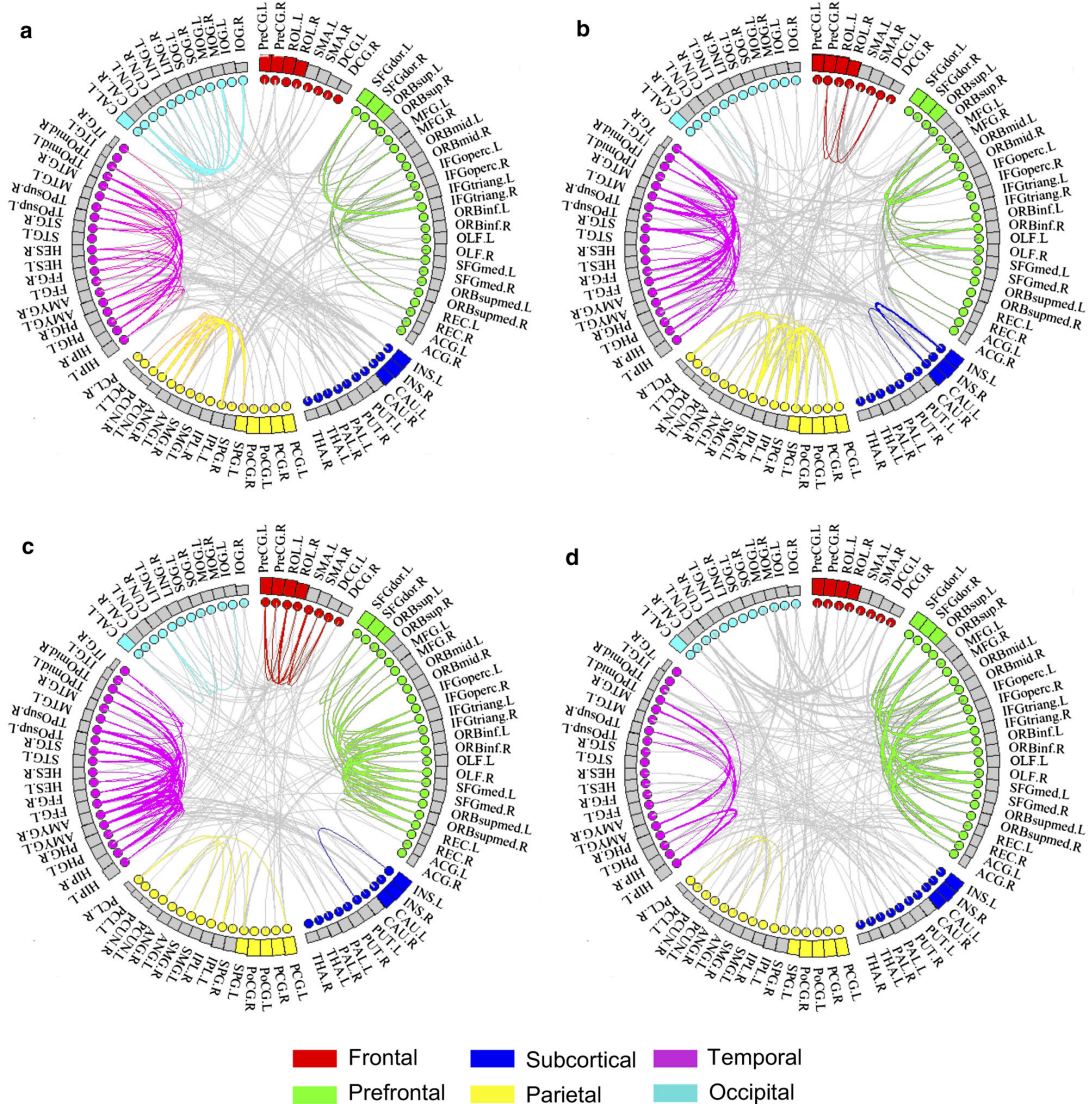
Connection patterns of state 1 (**a**), state 2 (**b**), state 3 (**c**) and state 4 (**d**). All brain regions were anatomically divided into six modules. Red represents the frontal lobe, green the prefrontal lobe, deep blue the subcortex, yellow the parietal lobe, purple the temporal lobe, and cyan the occipital lobe. The grey lines represent inter-module connectivities, and the rest of the lines represent intra-module connectivities

**Fig. 4:**
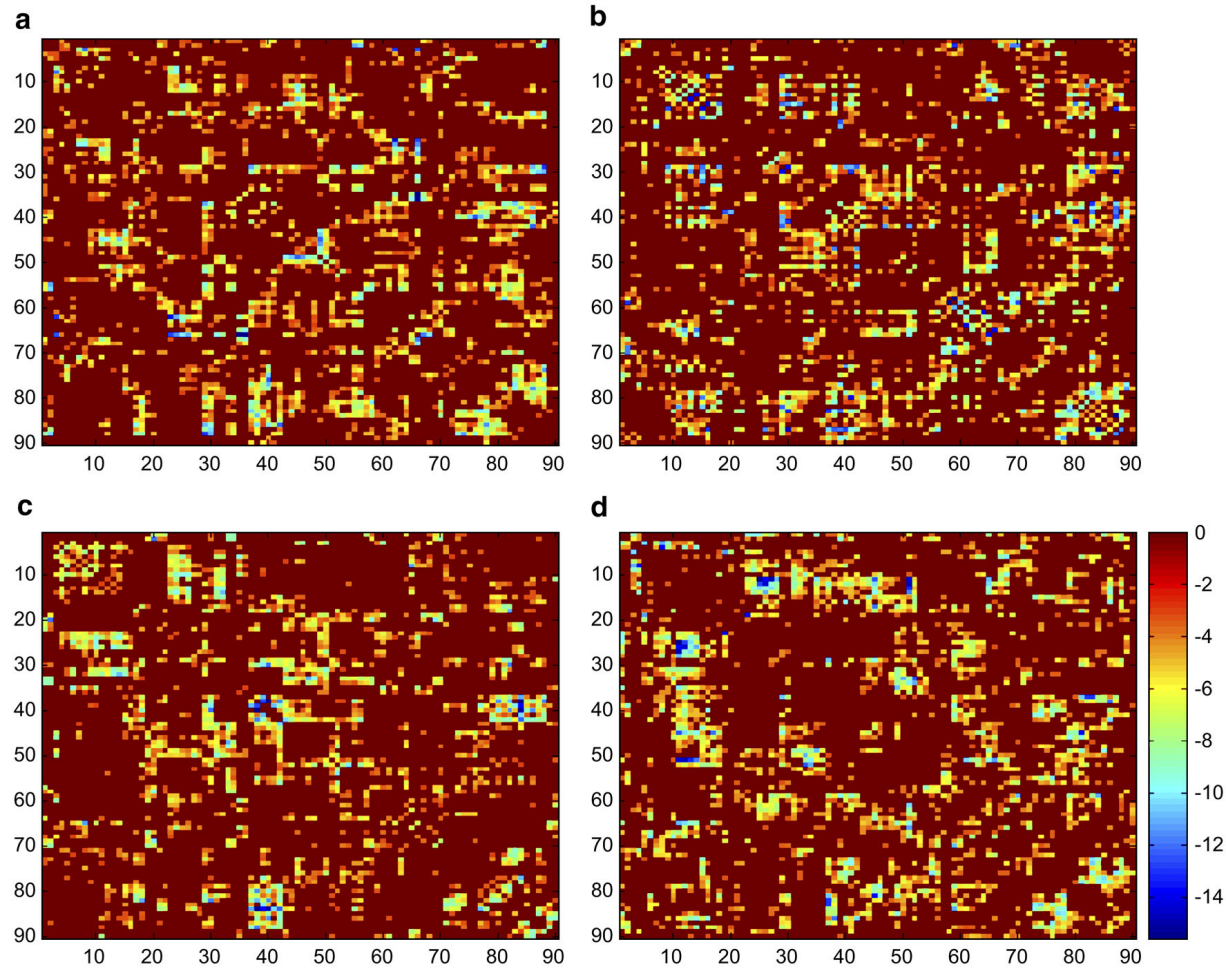
The matrix diagrams of specific differences in brain regions in state 1 (**a**), state 2 (**b**), state 3 (**c**), and state 4 (**d**)

All the brain regions were anatomically divided into six modules (Fig. 3). Figure 4 shows a matrix diagram of the specific differences in the brain regions. Compared with HCs, CSM patients showed positive coupling between the SMN and language network (LN), whereas the salience network (SN) and precuneus network (PN) showed decreased functional connectivity in state 1. In state 2, compared with HCs, patients with CSM showed decreased functional connectivity in the DMN, whereas positive coupling existed in the executive control network (ECN) and SN. In state 3, compared with HCs, CSM patients showed negative coupling between the right DMN and SMN, whereas the LN and auditory network (AN) showed positive coupling. In state 4, compared with HCs, the VN and SMN showed negative coupling, but the ECN showed increased functional connectivity.

### Topological properties of dynamic connectomics

**Fig. 5:**
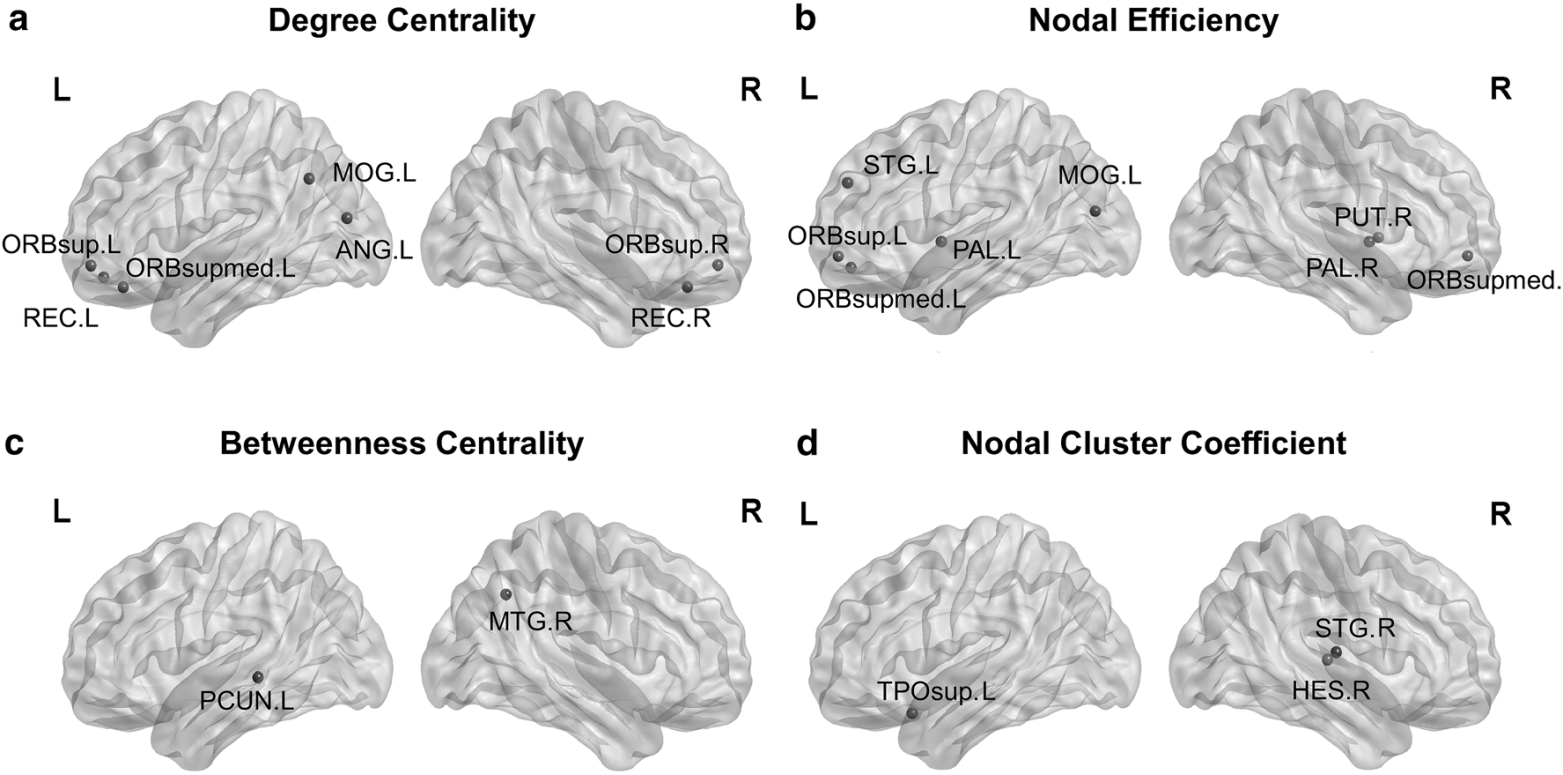
Between-group comparisons of degree centrality (**a**), nodal efficiency (**b**), betweenness centrality (**c**), and nodal cluster coefficient (**d**). The blue circles represent differences in topological properties in CSM patients compared with healthy controls

We found that patients with CSM showed a higher variance in local efficiency in the right Heschel gyrus, left and right superior temporal gyrus (both belonging to AN), and left middle temporal gyrus (belonging to LN). In contrast, there were no significant differences in the variance of global efficiency. Table 2 and Fig. 5 show the significantly different brain regions of degree centrality, nodal efficiency, betweenness centrality, and nodal cluster coefficient between the two groups.

**Table 2:** Abnormal topological properties in CSM patients as compared with healthy controls

Brain networks	Brain regions	FDR q values			
		Nodal degree	Nodal efficiency	Betweenness centrality	Nodal cluster coefficient
Auditory Network	STG.L	–	0.0286	–	
	STG.R				0.0054
	HES.R	–	–	–	0.0067
	TPOsup.L	–	–	–	0.0271
Language Network	MTG.R	–	–	0.0042	–
Dorsal attention network	ORBsup.L	0.0121	0.0327	–	–
	ORBsup.R	0.0251	–	–	–
Visual network	MOG.L	0.0489	0.0198	–	–
	MOG.R	–	0.0071	–	–
Basal ganglia network	PUT.L	–	0.0443	–	–
	PUT.R	–	0.0275	–	–
Executive control network	ORBsupmed.L	0.0028	0.0016	–	–
	ORBsupmed.R	–	0.0031	–	–
Default mode network	PCUN.L	–	–	0.0025	–
	PCUN.R	0.0042	–	–	–
	REC.L	0.0018	–	–	–
	ANG.L	0.0232	–	–	–

Regions were considered abnormal in CSM patients if they exhibited significant between-group differences (FDR q < 0.05)

*CSM* cervical spondylotic myelopathy; *STG.R* right superior temporal gyrus; *HES.R*, right heschl; *TPOsup.L* left temporal pole, superior temporal gyrus; *MTG.R* right middle temporal gyrus; *ORBsup.L* left superior frontal gyrus, orbital part; *ORBsup.R* right superior frontal gyrus, orbital part; *MOG.L* left middle occipital gyrus; *MOG.R* right middle occipital gyrus; *PUT.L* left lenticular nucleus, pallidum; *PUT.R* right lenticular nucleus, pallidum; *ORBsupmed.L* left superior frontal gyrus, medial orbital; *ORBsupmed.R* right superior frontal gyrus, medial orbital; *PCUN.L* left precuneus; *PCUN.R* right precuneus; *REC.L* left gyrus rectus; *ANG.L* left angular gyrus

## Discussion

In this study, we identified four different states by clustering all topography network maps across all sliding windows and subjects. We observed changes in the variability of network topological properties in dFC. Therefore, we discuss spontaneous dynamic reorganization in the whole brain of patients with CSM. Furthermore, we found that the variability in the network topological properties mainly focused on the DMN, LN, ECN, AN, and VN. In addition, we found that MDT in state 2 was significantly longer in the CSM group than in the HC group. According to previous studies, MDT means that subjects are primarily in the state of brain network connectivity patterns (Kim et al. 2017; Wang et al. 2019). This suggests that there is a longer dwell time pattern for state 2 in patients with CSM.

In state 1, patients with CSM showed positive coupling between the SMN and LN, whereas the SN and PN showed decreased functional connectivity. The SMN mainly regulates sensory and motor functions, and an abnormal connection of network functions corresponds to the clinical symptoms of patients. LN is important for maintaining sufficient language performance (Pistono et al. 2021).

As mentioned above, MDT in state 2 was significantly longer in the CSM group than in the HC group. This may indicate that patients with CSM are primarily in this state of brain function. The DMN and ECN are a pair of networks with opposite functions; they control cognitive and emotional executive functions to better internally self-focused attention of the central nervous system (Coppola et al. 2019; Davey et al. 2016; Qiao et al. 2020). The DMN supports internal psychological exploration and the ECN supports external stimuli and tasks. The switching of these two networks reflects the balance of conversion between internally and externally oriented cognition (Finn et al. 2015; Zou et al. 2021). We speculate that the increased functional connectivity of the ECN may be caused by the external stimulus of pain and stiffness in the neck in patients with CSM. The SN is mainly involved in attention, working memory, conflict monitoring, and salience detection (Alcauter et al. 2014; Steiner et al. 2020). The positive coupling between the ECN and the SN of patients with CSM may imply that external stimuli can enhance the coupling of these two networks within state 2. In this state, the brain mainly processes externally oriented cognition, attention, working memory, conflict monitoring, and salience detection.

In state 3, patients with CSM showed negative coupling between the DMN and SMN, whereas the LN and AN showed positive coupling. Prior studies have suggested that auditory expertise can enhance the perceptual categorization of speech (Bidelman 2015; Bidelman et al. 2014). Another recent study proposed that auditory experience modulates specific engagement and inter-regional communication between LNs and ANs (Bidelman and Walker 2019). Therefore, the positive coupling between LN and AN could promote the modulation of auditory and speech functions in patients with CSM.

In state 4, VN and SMN showed negative coupling, but ECN showed increased functional connectivity. VN plays a key role in the processing of visual movement, which can affect the acuity of visual space perception (Dole et al. 2013). Some researchers believe that the interaction between vision and sensorimotor function is crucial for movement control and motor learning (Glickstein 2000). Visual-sensorimotor interaction is important for movement control and motor learning (Greicius et al. 2003).

Compared to the HC group, the CSM group showed an increase in local efficiency. This may reflect abnormalities in local information transfer within the functional connectivity network in patients with CSM. In other words, the information transfer between critical nodes within a neighborhood tends to be more unstable and variable in patients with CSM. Some studies (Cohen 2018; Liao et al. 2014, 2017) have indicated that increased variability within dynamic brain networks may promote better integration of brain information and better flexibility of switching in cognitive processes. However, there were no significant differences in the global efficiency variance, which may indicate that information transfer in the whole brain is less affected, invariable, and remains stable. Besides, significant differences in variance also occurred in nodal efficiency, betweenness centrality, nodal degree, and nodal cluster coefficient. Interestingly, compared to HCs, variances in all nodal properties of time-varying brain connectivity were decreased in patients with CSM. These findings suggest that the brain areas of patients with CSM tend to be “fragile” and vulnerable to disturbance and damage, whereas the necessary resources may be recruited more quickly in the face of changing mission requirements in HCs. This finding is similar to the results of other neurological disorders, such as schizophrenia (Yu et al. 2015). For nodal efficiency, the significantly different brain regions were mainly located in the dorsal attention network, VN, ganglia network, AN, and ECN. For betweenness centrality, significantly different regions of the brain were mainly located in the DMN and LN. For nodal degree, significant differences in brain regions were mainly located in the ECN, DMN, dorsal attention network, and VN. For the nodal cluster coefficient, significantly different brain regions were mainly located in the AN. It was not difficult to observe that the brain regions with variance differences corresponded to the brain networks in the previous four states to some extent. Therefore, we have more sufficient reasons to believe that these networks play important roles in the pathogenesis of CSM.

This study had several limitations. First, it was a cross-sectional study that revealed patterns of dynamic connectivity of brain networks in patients with CSM. However, longitudinal studies are necessary to evaluate the effects of decompression surgery on alterations in the dynamic connectomics of brain networks. Second, this study included all patients with CSM. However, the dynamic brain network results in patients with mild, moderate, and severe CSM may differ. Finally, this study only analyzed the differences in the functional brain networks of patients with CSM. The topological properties of structural brain networks also require further exploration.

## Conclusion

In this study, we explored the alterations in the dynamic topography of whole-brain functional networks in patients with CSM. We used the K-clustering method to separate the brain network patterns of patients with CSM into four states and concluded that patients with CSM are mainly in state 2. Therefore, we further speculate that the brain mainly processes externally oriented cognition, attention, working memory, conflict monitoring, and salience detection in patients with CSM. Our study not only found abnormalities in brain networks but also supplemented previous studies on resting-state brain networks in patients with CSM. Dynamic functional connection states may offer new insights into intrinsic functional activities in the brain networks of CSM. In addition, the variance of topological organization of whole-brain functional networks may suggest instability of the brain networks in patients with CSM.

## Footnote

The authors are accountable for all aspects of the work and will ensure that any questions related to the accuracy or integrity of any part of the work are appropriately investigated and resolved. All figures and tables presented in this study are original and have not been previously published.

## Declarations

### Data availability

The data that support the findings of this study are available from the First Affiliated Hospital of Nanchang University, but restrictions apply to the availability of these data, which were used under license for the current study and so are not publicly available. However, the data are available from the authors upon reasonable request and with permission from the First Affiliated Hospital of Nanchang University.
